# A Novel Senescence-Specific Gene (*ZmSAG39*) Negatively Regulates Darkness and Drought Responses in Maize

**DOI:** 10.3390/ijms232415984

**Published:** 2022-12-15

**Authors:** Chunlai Wang, Bai Gao, Nannan Chen, Peng Jiao, Zhenzhong Jiang, Chunli Zhao, Yiyong Ma, Shuyan Guan, Siyan Liu

**Affiliations:** 1 College of Life Sciences, Jilin Agricultural University, Changchun 130118, China; 2 College of Agronomy, Jilin Agricultural University, Changchun 130118, China; 3 College of Forestry and Grassland, Jilin Agricultural University, Changchun 130118, China; 4 Joint International Research Laboratory of Modern Agricultural Technology, Ministry of Education, Jilin Agricultural University, Changchun 130118, China

**Keywords:** *ZmSAG39*, maize, leaf senescence, darkness stress, drought stress

## Abstract

The papain-like cysteine proteases (PLCPs) is a subfamily of cysteine proteases that plays an important role in leaf senescence, and some of its members are involved in the regulation of plant growth and development under stress. In this study, we cloned a new gene, *ZmSAG39*, from maize. Expression profile analysis showed that *ZmSAG39* was induced by darkness and drought treatments. In addition, the *ZmSAG39* overexpression in maize accelerated the senescence of maize leaves under darkness and drought treatments. However, the knockout of *ZmSAG39* in maize enhanced the resistance of maize to darkness and drought stresses and reduced the degree of senescence of maize leaves. Under drought stress, compared with WT plants, the knockout lines had a higher seed germination rate, seedling survival rate and chlorophyll content, and lower reactive oxygen species (ROS) level and malondialdehyde (MDA) content. In addition, quantitative real-time PCR (qRT-PCR) analysis showed that *ZmSAG39* negatively regulated some stress-related genes but positively regulated senescence-related genes under darkness and drought stress conditions. To summarize, these results indicate that *ZmSAG39* is a senescence-related gene and plays a negative role in response to darkness and drought stresses. This study laid a theoretical foundation for the innovation of maize germplasm resources with high quality, high yield and strong stress resistance.

## 1. Introduction

Maize (*Zea mays* L.) is one of the most widely planted crop globally and is also commonly used as raw material for feed [1]. The late growth stage of maize is a key period for yield formation. At this stage, premature initiation of plant senescence will shorten the lengths of the leaf function stage and the grain filling stage, resulting in reduced dry matter accumulation, which seriously affects the yield and quality of maize. Delaying senescence may enhance carbon assimilation, leading to increased grain and biomass yield [2,3]. Therefore, it is particularly important to cultivate maize varieties that can delay leaf senescence.

Leaf senescence is a complete dynamic process for plants to adapt to the external environment and is the last stage of growth and development [4]. When plants are under stress, some of their organs can avoid excessive consumption of nutrients through premature senescence, and then transport them to other organs to ensure the normal growth and development of the plants [5,6]. Senescence can be manifested in all parts of a plant. Leaves, as the main part of the plant involved in photosynthesis, are the most obvious place where plant senescence can be observed. The early symptom of premature senescence in leaves is the degradation of chlorophyll, protein, lipid, nucleic acid and other macromolecules, resulting in decreased photosynthesis and yellowing of leaves. In the middle and late stage, the leaves gradually turn brown, wither and fall off, and even the whole plant withers [7,8].

Papain-like cysteine proteases (PLCPs), one of the most abundant subfamilies of cysteine proteases, have nucleophilic cysteine thiols in their active sites and have been reported to be involved in the regulation of plant growth, senescence and abiotic stress [9]. At present, the cysteine protease gene family is generally divided into nine subfamilies, RD21, CEP, XCP, XBCP3, THI, SAG12, RD19, ALP and CTB9 [10]. The common protein structure of PLCP is a papain-like fold consisting of two domains (α-helix and β-fold domains). These are connected to each other, forming a deep fissure that acts as a substrate binding groove, and its interior contains the catalytic triad Cys-His-Asn, which is a conserved feature of PLCP [11]. PLCP is a very stable enzyme that is commonly found in environments where proteolysis is severe, such as apoplasmic bodies, vacuoles and lysosomes. To target these sites, PLCPs are encoded as preproproteins and carry various targeted signal peptides. Some PLCPs carry a signal at the C-terminus that can be retained in the endoplasmic reticulum (ER) (KDEL, HDEL) [12], whereas others carry a signal at the N-terminus of the proprotease for vacuolar targeting (NPIR) [13].

Most of the senescence-related cysteine proteases in the plant genome belong to the papain-like family, and they show different expression patterns during plant development [10]. Transcription products of some PLCP genes are down-regulated in senescent leaves, such as *NtCP2* in tobacco and *SEN102* in daylily [14]. However, most PLCP genes are up-regulated during leaf senescence. These PLCP genes include *NtCP1* in tobacco [15], *AtRD21A* and *SAG12* in Arabidopsis [16,17], *HbSAG12H1* in rubber [18] and *SCPC3* in sweet potato [19]. In addition, PLCP also responds to different stress conditions, including drought, darkness and pathogen infection. Crops usually suffer from insufficient light, drought and other adverse environmental factors during their growth and development, resulting in premature leaf senescence, reduced yield and quality. These adverse environmental factors make the stressed plants start the leaf senescence process in advance [20]. The early senescence in leaves is significantly correlated with the accumulation of reactive oxygen species (ROS) and the decrease in the activities of antioxidant enzymes such as superoxide dismutase (SOD), catalase (CAT) and peroxidase (POD) [21]. Drought stress in the grain filling period will affect the vegetative growth of plants and lead to an imbalance in the sink source ratio. Drought stress will accelerate leaf senescence and greatly reduce the supply of functional leaves and photosynthetic substances [20]. In a study where Barley *HvPap-1* was induced under darkness and nitrogen starvation conditions, HvPap-1 overexpression accelerated leaf senescence, while silencing of *HvPap1* delayed the senescence process [22]. In water-deficient barley leaves, cysteine protease inhibitors *HvCPI-2* and *HvCPI-4* delay the natural aging process and improve drought resistance by regulating the expression and activity of C1A proteases (*HvPap-1*, *HvPap-12* and *HvPap-1*6) [23]. *HpXBCP3* was involved in the regulation of cell growth, salt stress response and chlorophyll synthesis in microalgae. This may be due to the strict control of protease activity to avoid sudden protein degradation [9].

The CRISPR/Cas9 system is a gene-editing technology that makes modifications to genetic material inside cells. It has the advantages of a short experiment period, high efficiency, non-toxicity and being economical, so it is widely used in plant gene editing [24]. After *OsGRP3* gene knockout by CRISPR-Cas9 technology, the results showed that the decrease in POD enzyme content led to the decrease in lignin content and the increase in H_2_O_2_ content in the *Osgrp3* knockout line. Therefore, *OsGRP3* positively regulated lignin biosynthesis and H_2_O_2_ scavenging by PODs to enhance drought resistance in rice [25]. CRISPR/Cas9-generated *OsPYL9* mutants have potential to improve both drought tolerance and the yield of rice [26]. In this study, CRISPR-Cas9 technology was used to analyze the gene function.

At present, there are few studies on PLCP genes in maize. In this study, we cloned a new senescence-related gene, *ZmSAG39*, from maize plants and analyzed its molecular characteristics. Secondly, the subcellular localization of *ZmSAG39* protein was investigated. Then, yeast two-hybrid experiments were performed to identify proteins that might interact with it. Finally, through abiotic stress analysis, the results indicated that *ZmSAG39* may be involved in the senescence of maize leaves and enhance the sensitivity to darkness and drought stresses.

## 2. Results

### 2.1. Cloning and Analysis of the ZmSAG39 Gene

**Figure 1:**
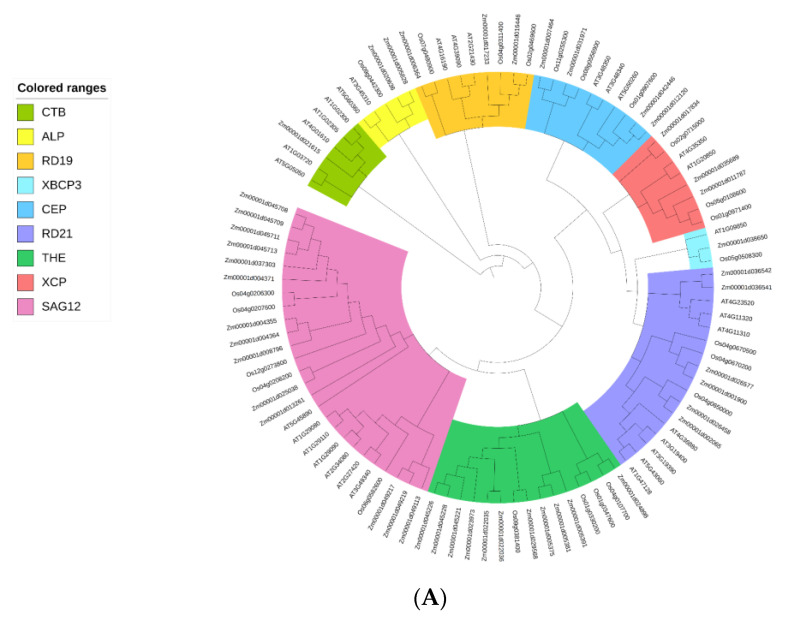

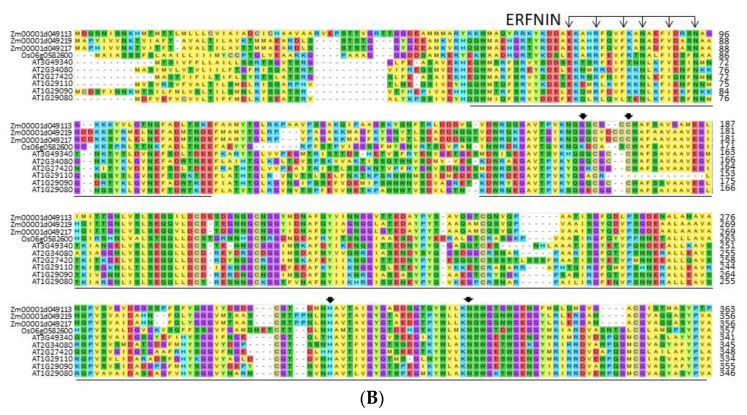
Protein sequence analysis and phylogenic tree analysis of *ZmSAG39*. (**A**) Phylogenetic analysis of Papain-like cysteine proteases (PLCP)-encoding enzymes from *At (Arabidopsis thaliana)*, *Os (Oryza sativa)* and *Zm (Zea mays)*. A neighbor-joining tree was generated using MEGAX with 1000 bootstrap replicates. Different background colors showed different subgroups. (**B**) Multiple sequence alignment of predicted protein sequences belonging to a subgroup of 10 closely related PLCPs. C1A (papain) domain is highlighted by lines. ERFNIN motif is highlighted by continuous arrows. The catalytic triad Cys, His and Asn and also the Glu active site residue are indicated by the arrows.

A 1092 bp CDS (coding sequences) region (LOC100274134) was cloned from a maize genome database and named *ZmSAG39*. The DNA sequence of *ZmSAG39* was located in chromosome 4 NC_050099.1 (16651845-16653321). *ZmSAG39* encodes 363 amino acids with a molecular weight of 38.9 kD. Through a genome-wide search, 45, 35 and 24 PLCPs were identified in maize, Arabidopsis and rice, respectively. The phylogenetic tree analysis showed that *ZmSAG39* belongs to the SAG12 subclass of papain-like cysteine proteases (C1A) (Figure 1A) [27,28]. Multiple sequence alignment of the 10 PLCPS in this subgroup showed that *ZmSAG39* encodes a cysteine protease, which contains C1A (papain) domain, including protease inhibitor I29 and protease C1 (autoinhibitory domain and catalytic domain). In addition, the protease contains the highly conserved ERFNIN motif. The four active sites of this protease are Glu (G169), Cys (C175), His (H310) and Asn (N331). The conserved catalytic triad Cys-His-Asn was found at similar positions to those of other PLCPs (Figure 1B).

### 2.2. Stress-Induced Expression Profiles of ZmSAG39

**Figure 2:**
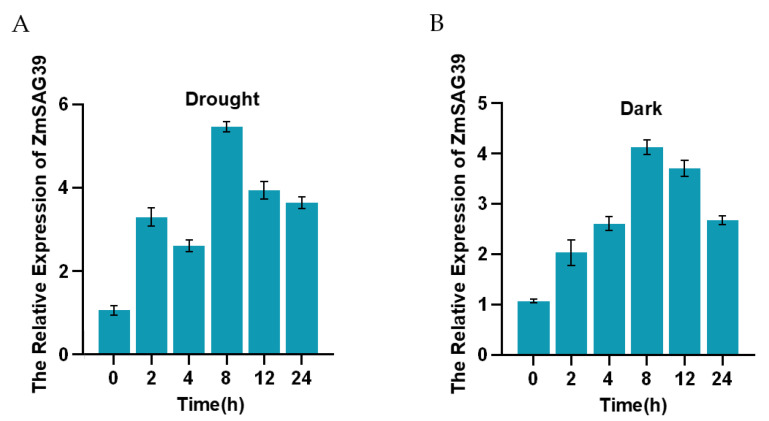
Response of *ZmSAG39* to abiotic stress. (**A**,**B**) Expression patterns of *ZmSAG39* in maize leaves under dark and drought stress conditions. Data are shown as mean ± SD from three independent experiments.

We analyzed the effect of abiotic stress on the expression of *ZmSAG39*. This study found that both darkness and drought stresses can significantly increase the expression of *ZmSAG39*, which was 5.57-fold and 4.29-fold higher than those without treatment, respectively (Figure 2A,B). These results indicated that *ZmSAG39* played an important role in stress-induced leaf senescence.

### 2.3. ZmSAG39 Reduces Darkness Tolerance in Maize

**Figure 3:**
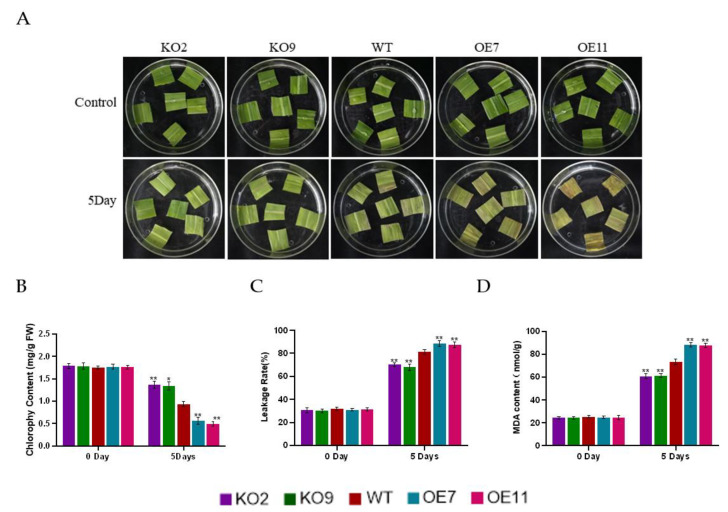
Dark stress tolerance test in the wild-type (WT) and transgenic plants. (**A**) Leaf senescence phenotype of WT and transgenic lines after dark treatment. (**B**–**D**) Total chlorophyll content, ion leakage rate and malondialdehyde (MDA) content of different genotypes. Using Student’s *t*-test, asterisks indicate statistically significant differences (* *p* < 0.05; ** *p* < 0.01). Data are shown as mean ± SD from three independent experiments.

To determine phenotypic differences between wild-type (WT), knockout and overexpression lines under darkness stress, detached leaves of three-week-old WT, knockout and overexpression plants were treated in complete darkness for 5 days. After 5 days of darkness treatment, we observed that the overexpression lines exhibited a more severe senescence phenotype than the WT and knockout plants (Figure 3A). In addition, the chlorophyll content of the overexpression lines was always lower than that of the WT and knockout lines, and the membrane ion leakage rate and malondialdehyde (MDA) content were always higher than those of the WT and knockout lines (Figure 3B–D). These data suggest that overexpression of *ZmSAG39* accelerates the rate of darkness-induced senescence and increases susceptibility to darkness.

### 2.4. ZmSAG39 Reduces Drought Tolerance in Maize

**Figure 4:**
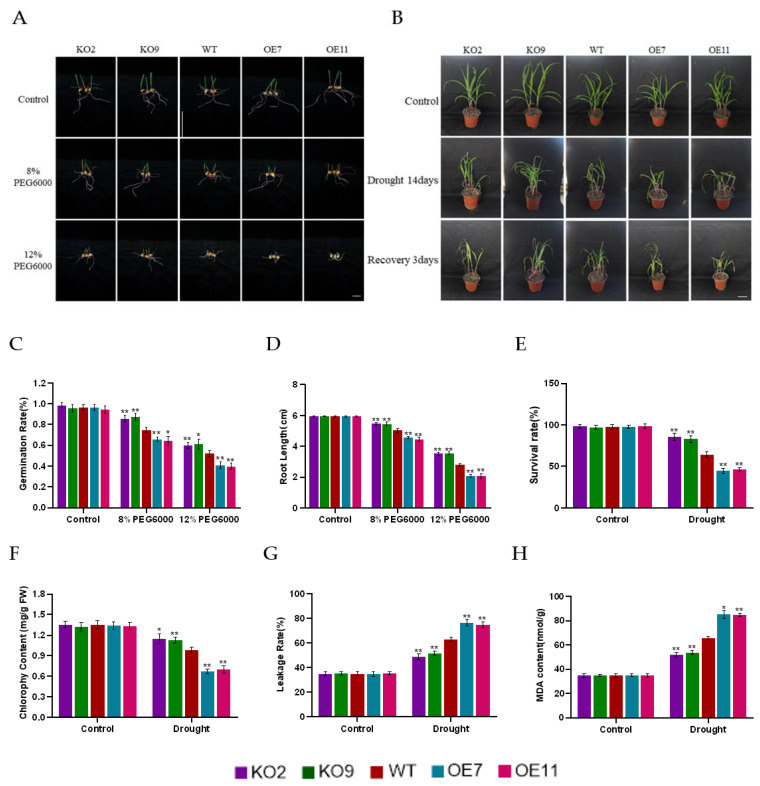
Drought stress tolerance test in the WT and transgenic plants. (**A**) Seeds of WT and transgenic lines germinated on filter paper soaked in different solutions of PEG6000: water without PEG6000; 8% PEG8000; 12% PEG6000; (**B**) Leaf senescence phenotype of WT and transgenic lines after drought treatment. Scale bar = 1 cm. (**C**,**D**) Statistics of germination rate and root length for different genotypes. (**E**) Survival rate of WT and transgenic plants after re-watering for 3 days. (**F**–**H**) After treatment with drought for 14 days, the total chlorophyll content, ion leakage rate and MDA content of isolated leaves of different genotypes. Using Student’s *t*-test, asterisks indicate statistically significant differences (* *p* < 0.05; ** *p* < 0.01). Data are shown as mean ± SD from three independent experiments.

The germination rates of transgenic and WT seeds were preliminarily verified under 8% and 12% PEG6000 treatment, respectively. The results showed that the germination rate and the root length of the knockout lines were significantly higher than those of the WT and overexpression lines under the two concentration conditions (Figure 4A,C,D). These results indicated that *ZmSAG39* knockdown was more beneficial to seed germination.

To confirm the role of *ZmSAG39* in maize drought response, transgenic and WT maize were sown in soil and then subjected to water stress experiments. After 2 weeks of drought stress, maize seedlings grew slowly. *ZmSAG39* overexpression lines showed more severe wilting phenotype than WT plants, and leaf tips turned yellow and significantly curly. Compared with WT, *ZmSAG39* knockout lines showed a more upright phenotype, and leaves remained mostly green and slightly curly (Figure 4B). In order to test whether the dehydration phenotype could be recovered, a rehydration experiment was carried out. After 3 days of rehydration, the knockout plants had grown fresh new leaves, and about 70% of them had resumed growth; about 55% of WT plants recovered growth. However, only about 25% of overexpression plants were able to resume growth after rehydration (Figure 4E). In addition, the membrane ion leakage rate, chlorophyll content and MDA of WT and transgenic plants were analyzed. The results showed that the membrane ion leakage rate and MDA content of overexpression plants were higher than those of WT and knockout plants, and the chlorophyll content was lower than that of WT and knockout plants (Figure 4F–H). These results suggest that overexpression of *ZmSAG39* accelerates drought-induced senescence and increases susceptibility to drought, whereas knockout of *ZmSAG39* contributes to drought resistance.

### 2.5. Expression of Related Genes Were Altered in ZmSAG39 Transgenic Plants

**Figure 5:**
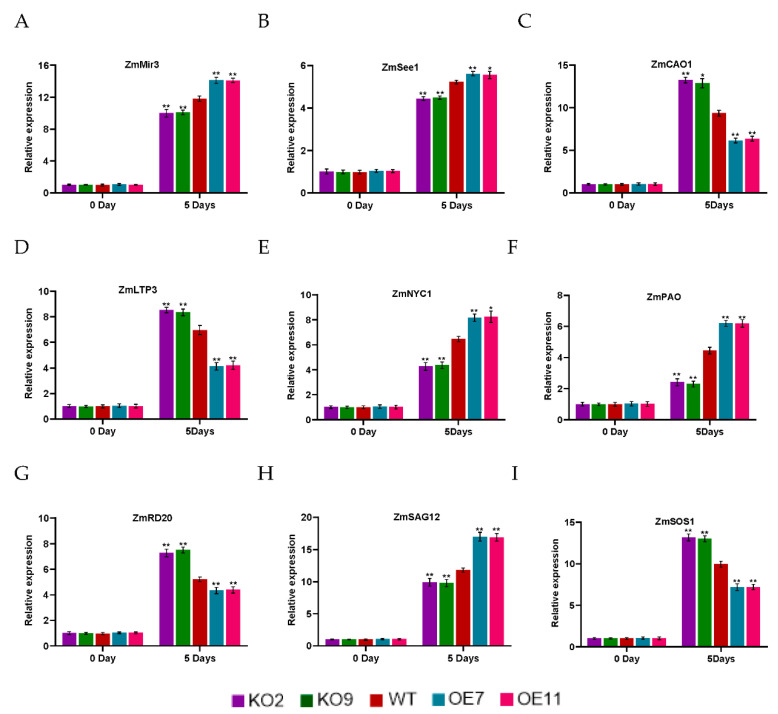
Expression levels of related genes in WT and transgenic plants under normal condition and dark treatment. Three-leaf stage maize leaves were treated in dark for 5 days. The expression levels of related genes during dark stress were analyzed by qRT-PCR. (**A**–**I**) Expression analysis of stress-related genes, Chlorophyll-related genes and senescence-related genes. The expression level was normalized to that of maize *ZmACTIN1*. Using Student’s *t*-test, asterisks indicate statistically significant differences (* *p* < 0.05; ** *p* < 0.01). Data are shown as mean ± SD from three independent experiments.

**Figure 6:**
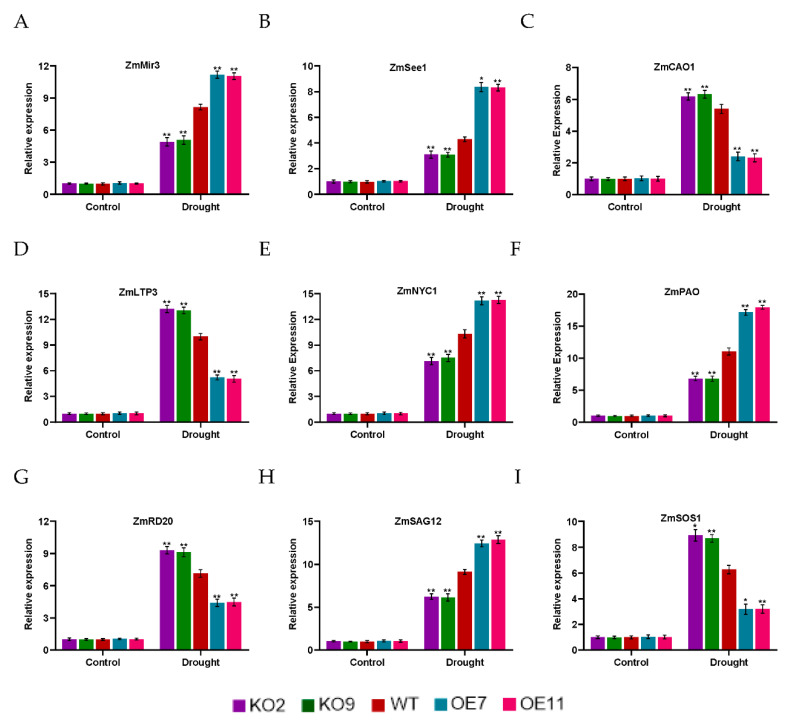
Expression levels of related genes in WT and transgenic plants under normal condition and drought treatment. Three-leaf stage maize seedlings were withheld in water for 7 days. The expression levels of related genes during drought stress were analyzed by qRT-PCR. (**A**–**I**) Expression analysis of stress-related genes, Chlorophyll-related genes and senescence-related genes. The expression level was normalized to that of maize *ZmACTIN1*. Using Student’s *t*-test, asterisks indicate statistically significant differences (* *p* < 0.05; ** *p* < 0.01). Data are shown as mean ± SD from three independent experiments.

To further understand the regulatory mechanism of *ZmSAG39* in response to darkness stress, we analyzed the expression levels of several known stress-responsive genes, *ZmSOS1*, *ZmLTP3* and *ZmRD20*, in WT and transgenic plants under control and stress conditions. *RD20* is often used as a stress marker gene and plays an important role in plant stress resistance [29]. Chlorophyll-related genes (*ZmNYC1*, *ZmPAO*, *ZmCAO1*) and senescence-related genes (*ZmSAG12*, *ZmSee1*, *ZmMir3*) were also analyzed. After darkness stress treatment, the expression levels of all stress-related genes in the knockout plants were higher than those in WT and overexpression lines. The expression of chlorophyll synthesis genes *ZmCAO1* was highest in the knockout plants, while the expression of chlorophyll degradation genes *ZmNYC1* and *ZmPAO* was lowest in the knockout plants. The expression levels of all senescence-related genes were highest in the overexpression plants. After drought stress treatment, there was a similar pattern of stress-response gene activation in knockout plants (Figure 5). Under the control condition, the expression levels of the analyzed genes in the transgenic plants were similar to those in WT to some extent. However, under drought stress, the expression levels of stress-related genes and chlorophyll synthesis-related genes in the knockout plants were significantly higher than those in the WT plants, while the expression levels of senescence-related genes and chlorophyll degradation-related genes were lower than those in WT (Figure 6). These results indicated that under darkness stress and drought stress, *ZmSAG39* overexpression had a negative regulatory effect on these stress-related genes and chlorophyll synthesis-related genes, while it had a positive regulatory effect on senescence-related genes and chlorophyll degradation-related genes.

### 2.6. Monitoring of ROS Content and Antioxidant Enzymes’ Activities in Transformed Lines under Drought Stress

**Figure 7:**
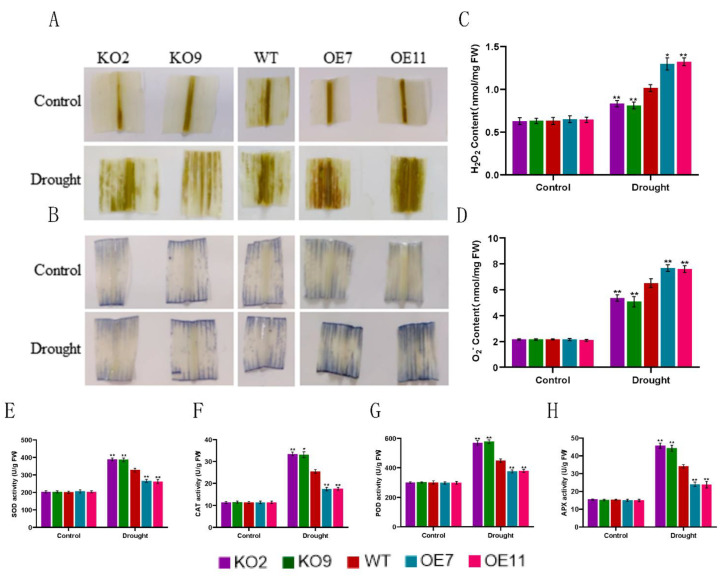
*ZmSAG39* can promote the accumulation of reactive oxygen species (ROS). (**A**) Diaminobenzidine (DAB) staining. Maize leaves were stained with DAB after drought treatment. (**B**) Nitro-blue tetrazolium chloride (NBT) staining. Maize leaves were stained with NBT after drought treatment. (**C**,**D**) Accumulation of H_2_O_2_ and O_2_^−^ in leaves of different lines. (**E**–**H**) Analysis of superoxide dismutase (SOD), catalase (CAT), peroxidase (POD) and ascorbic acid peroxidase (APX) activity. Using Student’s *t*-test, asterisks indicate statistically significant differences (* *p* < 0.05; ** *p* < 0.01). Data are shown as mean ± SD from three independent experiments.

Plants are vulnerable to environmental stress in all aspects of growth and development, which can cause serious damage to plant survival and production. Among these environmental factors, drought stress is one of the most important challenges limiting crop growth and grain yield [2,30]. On the one hand, drought stress causes dehydration, which leads to ion stress and osmotic stress, thus destroying the dynamic balance of cells. On the other hand, drought stress induces oxidative stress, which produces a large number of reactive oxygen species (ROS) that cause membrane lipid oxidation, protein and DNA damage, leading to plant wilting and even death [31]. In this study, the presence of ROS was monitored by DAB and NBT staining. After 7 days of drought treatment, DAB and NBT staining was more obvious in the leaves of *ZmSAG39* overexpression lines compared with WT leaves (Figure 7A,B), and the leaves of knockout lines showed lighter staining. Similar to the phenotype, H_2_O_2_ and O_2_^−^ content also largely accumulated in overexpression lines but less in knockout lines (Figure 7C,D). Antioxidant enzymes such as peroxidase, catalase and superoxide dismutase can provide basic protection for cells and remove ROS accumulated in plants to protect them from damage. Under drought stress, SOD, CAT APX and POD enzyme activities of the knockout plants were higher than those of the WT, while those of the overexpression transgenic plants were lower than those of the WT (Figure 7E–H). This also makes the leaves of overexpression strains senesce faster than those of knockout strains.

### 2.7. Subcellular Localization and Yeast Two-Hybrid Test of ZmSAG39 Protein

**Figure 8:**
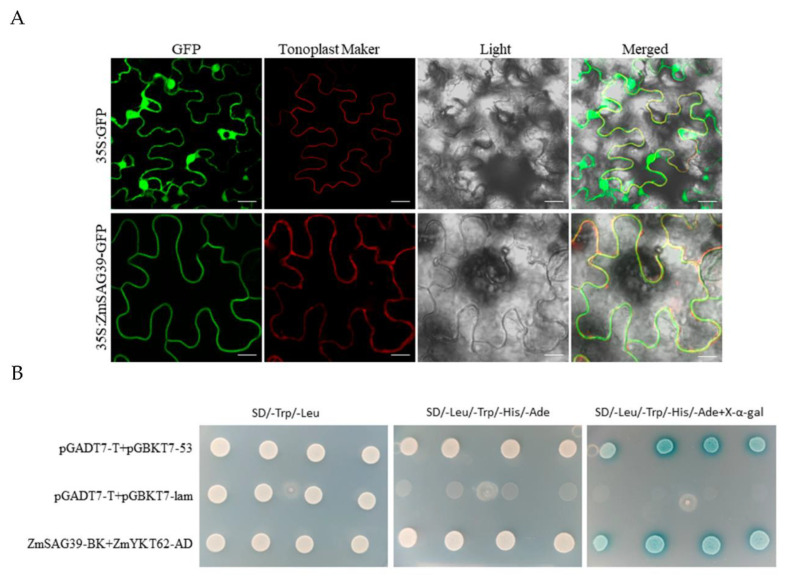
Subcellular localization and yeast two-hybrid test of ZmSAG39 protein. (**A**) Subcellular localization analysis of ZmSAG39 proteins in tobacco cells. The scale bar represent 50 μm. (**B**) Interaction testing between pGBKT7-ZmSAG39 and pGADT7-GmYKT62. Positive control: pGADT7-T +pGBKT7-53; negative control: pGADT7-T+pGBKT7-Lam.

In order to clarify the subcellular localization of *ZmSAG39* protein, Agrobacterium tumefaciens EAH105 strains containing 35S:ZmSAG39-GFP and 35S:GFP (control) were inoculated into tobacco epidermal cells for transient expression. The green fluorescence signal of 35S:GFP empty vector was distributed throughout the cell, while 35S:ZmSAG39-GFP only showed green fluorescence on the membrane. The superposition field showed that the green fluorescence and the red fluorescence of *ZmSAG39* protein could coincide well, indicating that *ZmSAG39* was distributed on the vacuoles’ membrane (Figure 8A).

pGADT7-YKT62 and pGBKT7-ZmSAG39 were co-transformed into a Y2HGold yeast competent state to verify their interaction. The results showed that pGADT7-YKT62 and pGBKT7-ZmSAG39 were transformed into four deficient plates (containing X-α-Gal); the yeast strain grew well and showed a blue color, indicating that the proteins expressed by pGADT7-YKT62 and pGBKT7-ZmSAG39 interact with each other in yeast (Figure 8B).

## 3. Discussion

In this study, we isolated a PLCP gene from the maize genome and named it *ZmSAG39*. We identified 45 members of PLCP genes from the maize genome. The evolutionary divergence of PLCP genes showed their classification into nine subfamilies, consistent with studies on other plant species (Figure 1A) [10]. *ZmSAG39* protease contains two conserved domains, protease inhibitor I29 and protease C1, and has a conserved catalytic triad Gln/Cys-His-Asn sequence alignment. Phylogenetic analysis showed that *ZmSAG39* belongs to the papain-like subfamily and has high homology to PLCP proteins known from other plants (Figure 1B). In this study, we found that *ZmSAG39* was located at the vacuole membrane (Figure 8A), which is roughly consistent with the localization of *CaCP* in pepper [32].

Based on previous studies, this study found that the PLCP gene plays an important role in a variety of biological and abiotic stresses. In sweet potato, *SPCP2* is a senescence-related gene and its expression pattern is enhanced during natural leaf senescence and stress induction [33]. The expression of the Glyma.14G085800 gene in soybean increased significantly after drought treatment [34]. Consistent with previous results, the qRT-PCR analysis in this study shows that *ZmSAG39* responds to both darkness and drought treatments, indicating that *ZmSAG39* can respond to various abiotic stresses (Figure 2). It was found that the overexpression of *ZmSAG39* led to early senescence of maize leaves under dark conditions (Figure 3A). Under drought stress, the seed germination rate of *ZmSAG39* overexpression plants was lower than that of WT and knockout lines, and seriously affected the growth and development of root length (Figure 4C,D). At the same time, the seedling survival rate of overexpression strains was significantly lower than that of WT and knockout lines (Figure 4F). These data indicate that *ZmSAG39* plays an active role in darkness- and drought-induced leaf senescence. In maize, transgenic plants with *ZmSAG39* knockout have higher stress resistance. These are consistent with previous research. For example, *CaCP* in pepper plants can be largely induced during leaf senescence. Loss of function of *CaCP* using the virus-induced gene-silencing technique led to enhanced tolerance to salt-induced and osmotic-induced stress [32]. Transgenic plants inhibited by *CtCP1*-anti showed higher tolerance to low temperature than WT and *CtCP1*-OE plants [35]. The overexpression of sweet potato *SPCP3* in Arabidopsis conferred sensitivity to drought stress [36]. In addition, the physiological and biochemical indexes related to resistance of *ZmSAG39* transgenic lines and WT were measured. First, the ion leakage rate and MDA content are important indicators for evaluating the damage of the plant cell membrane caused by osmotic pressure [37,38]. Phenotype results of overexpression of maize *ZmASR3* gene in Arabidopsis showed lower malondialdehyde (MDA) levels and higher relative water content (RWC) than the wild type under drought conditions, demonstrating that *ZmASR3* can improve drought tolerance. After water deficit treatments, leaves of *TaSNAC11-4B* overexpression lines displayed significantly higher membrane leakage rates than that of Col-0, demonstrating that expression of *TaSNAC11-4B* led to higher sensitivity to drought stress [39]. Based on these findings, our analysis indicated that the ion leakage rate and MDA content of *ZmSAG39* overexpression lines were higher than those of WT under drought and dark conditions (Figure 3C,D and Figure 4G,H). The above results were consistent with previous studies in Arabidopsis. Overexpression of *ZmSAG39* in Arabidopsis also showed increased sensitivity to drought and dark stress [40].

To adapt to various abiotic stresses, especially darkness and drought, plants have developed different response strategies, including regulating the expression of stress-response genes [41]. In this study, we found that the expression levels of stress-response genes *ZmSOS1*, *ZmLTP3* and *ZmRD20* in the knockout lines were higher than those in the control plants under stress treatment conditions, while the expression levels of these stress-response genes in the overexpression plants were significantly lower than those in the WT plants (Figure 5 and Figure 6). These results indicate that *ZmSAG39* negatively regulates darkness and drought stress responses, perhaps through the repression of stress-response genes. In general, plants can overproduce reactive oxygen species (ROS), which leads to cell damage by oxidizing DNA and proteins under abiotic stresses [42]. Therefore, cells have evolved an antioxidant mechanism to remove excess reactive oxygen species produced in the body, maintain the stability of redox status in cells and prevent damage to plants. In plants, enzymatic scavenging systems, including superoxide dismutase (SOD), catalase (CAT), ascorbic acid peroxidase (APX) and peroxidase (POD), play a crucial role in the removal of intracellular ROS [43,44]. In this study, under drought treatment, we were able to establish that overexpression plants accumulated more reactive oxygen species through DAB and NBT staining results (Figure 7A,B). The results of H_2_O_2_ and O_2_^−^ quantitation were consistent with the chemical staining data. These results show that the expression of *ZmSAG39* can promote the accumulation of ROS (Figure 7C,D). The SOD, CAT, POD and APX enzyme activities of *ZmSAG39* overexpression plants were lower than those of knockout and WT plants (Figure 7E–H). Knockout *ZmSAG39* is beneficial to the ROS dynamic balance of plants under stress.

The regulation of gene expression is a complex process, which is realized by the interaction between different proteins. Previously, we predicted that *ZmSAG39* might interact with the maize VAMP-like protein YKT62 through protein-interaction software (https://string-db.org/) (accessed on 1 December 2021). Then, we verified whether *ZmYKT62* interacted with *ZmSAG39* through the yeast two-hybrid technique. The results showed that *ZmYKT62* interacted with *ZmSAG39* (Figure 8B). *ZmYKT62* is a vesicle-associated membrane protein belonging to the synaptobrevin family and contains the R-SNARE conserved domain [45]. Studies have shown that *GsVAMP72* overexpression of soybean in Arabidopsis can change the ion content and down-regulate the expression of stress-response genes, thus significantly reducing the salt tolerance of Arabidopsis [46]. Therefore, we hypothesized that *ZmYKT62* may also play a role in abiotic stress. In future, we will further verify the specific functions of *ZmYKT62*.

In conclusion, our results showed that the expression level of *ZmSAG39* changed under drought stress, and the leaf senescence degree of *ZmSAG39* overexpression plants was significantly higher than that of WT and knockout lines. Knockdown of the *ZmSAG39* gene could significantly enhance the tolerance to drought stress and reduce the accumulation of ROS. At the same time, leaf senescence of *ZmSAG39* knockout transgenic plants was relatively slow compared with WT and overexpression lines under dark treatment. Therefore, the introduction of the *ZmSAG39* gene can effectively regulate the ability of maize to respond to abiotic stress, thus delaying leaf senescence.

## 4. Materials and Methods

### 4.1. Plant Materials and Growth Conditions

The maize M8186 inbred line was used as the material. Every four maize seeds were sown in the same plastic pot (20 cm × 25 cm) with sterilized soil (forest soil:Vermiculite 1:1), irrigated with water and sprayed with nutrient solution (P:N) once a week, and cultured in a greenhouse at 25 °C/16 °C (day/night temperature) with a photoperiod of 14 h light/8 h dark under 65% relative humidity.

### 4.2. Expression of ZmSAG39 under Abiotic Stress Treatments

Leaves detached from 3-week-old maize plants were treated with abiotic stress. Some leaves were transferred to a closed petri dish containing 20%PEG-6000 for culture, and the remaining leaves were cultured in the dark. The leaf samples were collected at 0, 4, 8, 12 and 24 h, respectively, frozen with liquid nitrogen and then stored in a refrigerator at −80 °C for later use.

### 4.3. Generation of Constructs and Transgenic Plants

In order to obtain the *ZmSAG39* overexpression constructs, PCR technology was used to amplify *ZmSAG39* using the cDNA of M8186 as template and primer SAG39-F/R, and then, it was subcloned into the BglII and BstEII restriction sites of pCAMBIA3301 vector (Supplemental Figure S1). Design the target sequence using the CRISPR-P website (http://crispr.hzau.edu.cn/CRISPR2/) (accessed on 4 February 2021): CCAGGTATTCAAGGCGAACGCGG, then the Biogle website (http://www.biogle.cn/index/excrispr) (accessed on 19 March 2021) was used to design the Oilgo primer, and the Oligo dimer was synthesized. Synthesis system: Buffer Anneal 18 μL, Low /UP Oligo 1 μL, ddH_2_O 5 μL. The Oligo dimer was then linked to the pCXB053 gene editing vector. Connection system: CRISP/Cas9 vector 2 μL, Oligo dimer 1 μL, Enzyme Mix 1 μL and ddH_2_O 6 μL. The constructs (CaMV35S::ZmSAG39 and gene-edited *ZmSAG39*) were converted into the *Agrobacterium* tumefaciens GV3101 and then transformed into maize callus [47]. The expression of the *ZmSAG39* gene in T2 generation plants was detected by qRT-PCR. In order to identify the mutations of the positive plants, the target gene was amplified by PCR with specific primers, cloned into the pMD18-T vector and sent to Jilin Kumei Biological Company for sequencing. Finally, the sequencing results were compared to analyze the target site mutations. Through the above analysis, two independent lines with *ZmSAG39* overexpression (OE7, OE11) and two independent lines with gene-edited *ZmSAG39* (KO2, KO9) were screened. Homozygous T2 transgenic plants were used for subsequent experiments (Figures S2 and S3). The primers used in this study are listed in Table S1.

### 4.4. Stress Treatments

For the darkness stress experiment, the detached leaves of maize plants grown in the culture chamber for 3 weeks were cut into 1 cm length and cultured in 3 mM MES buffer (pH 5.8), with the back side up, and treated in complete darkness at 28 °C for 5 days. Finally, the phenotypic changes were recorded.

For seed germination assays under drought treatment, the maize seeds of each strain (WT, OE7, OE11, KO2 and KO9) were individually loaded into 3 petri dishes with filter paper, making a total of 15 dishes, and the upper layer was also covered with filter paper. The petri dishes were divided into 3 groups, and each group had wild-type (WT), overexpression and knockout seeds. An appropriate amount of sterile water was poured into the plates of the first group as a control, 8%PEG6000 treatment solution was poured into the second group and 12%PEG6000 treatment solution was poured into the third group. Germination was counted after 3 days of germination. In addition, the root lengths of each line were measured.

For the drought tolerance experiments, WT and transgenic seedlings at the three-leaf stage were not watered for 14 days until the plants withered. The recovery of WT and transgenic lines was recorded after 14 days of drought and 3 days of rehydration. The plant survival rate was measured after rehydration for 3 days under normal conditions [48].

### 4.5. Multiple Alignments and Bioinformatic Analyses

The whole-genome data of maize sequences were downloaded from maizeGDB. The whole-genome data of rice and Arabidopsis thaliana sequences were downloaded from Ensembl. The molecular weight was estimated using ExPASy (http://web.expasy.org/compute_pi) (accessed on 1 May 2022). The phylogenetic analysis of *ZmSAG39* and cysteine protease from other species was aligned with CLUSTAL W using default parameters. A phylogenetic tree was constructed using MEGA version 7 from CLUSTAL W alignments. The neighbor-joining method was used to construct the tree.

### 4.6. NBT (Nitro-Blue Tetrazolium Chloride) Staining Assay

The NBT staining solution (0.5 gNBT, 500 μL sodium azide solution and 500 μL 1 M sodium phosphate buffer diluted with deionized water to 50 mL) was configured and loaded into a 50 mL centrifuge tube. The treated maize leaves were placed in a 50 mL centrifuge tube containing NBT staining solution and allowed to stand at room temperature for 60 min. Then, the NBT staining solution was dumped, 95% ethanol was added, and the samples were immersed. The centrifuge tube was placed in a water bath at 80 °C for decolorization. After all the green color of the samples faded, the samples were removed and the staining results were recorded.

### 4.7. DAB (3,3′-Diaminobenzidine) Staining Assay

The DAB staining method was performed as follows: DAB staining solution (20 mg DAB and 38 mL ddH20, PH adjusted with 0.2 M HCl, the final concentration was 0.5 mg/mL) was prepared and loaded into a 50 mL centrifuge tube. The leaves were immersed in DAB staining solution. The samples were incubated overnight at room temperature and decolorized with 95% ethanol in a water bath at 80 °C. After all the green color of the samples faded, the samples were removed and the staining results were recorded.

### 4.8. Determination of Physiological and Biochemical Activities

The contents of H_2_O_2_ and O_2_^−^ were determined as previously described [49,50]. The peroxidase (POD) activity was measured according to the previously described method [51], but with some modifications. The mixture contained 50 mM potassium phosphate buffer (pH 7.0), 0.1 mM EDTA, 50 mM guaiacol, 2% (*v*/*v*) H_2_O_2_, and 0.1 mL of the enzyme extract. Changes in the absorbance were measured at 470 nm [35]. The catalase (CAT) activity was analyzed in 3 mL reaction mixture (50 mM potassium phosphate buffer (pH 7.0), 2 mM Na_2_-EDTA, 0.1 mol/L H_2_O_2_ and 0.2 mL of enzyme extract), and the changes in the absorbance at 240 nm were measured over a period of 1 min. The superoxide dismutase (SOD) activity was analyzed in 3 mL reaction mixture (50 mM potassium phosphate buffer (pH 7.0), 130mM Met, 0.1 mM EDTA, 750 μM NBT, 60 μM riboflavin and 0.2 mL of enzyme extract). One unit of SOD activity was defined as the quantity of enzyme required to induce 50% inhibition of the NBT reduction measured at 560 nm [52]. The ascorbate peroxidase (AXP) activity was analyzed in a 3 mL reaction mixture containing 50 mM potassium phosphate buffer (pH 7.0), 0.5 mM ASA, 6 mM H_2_O_2_ and 50 µL of enzyme extract, and the changes in the absorbance at 290 nm were measured over a period of 30 s [53]. MDA was detected using the detection kit [54].

Maize leaves (0.2 g) were placed in a mortar and thoroughly ground with 80% acetone, then transferred to test tubes (3–4 mg of tissue in 1 mL of acetone). The tubes were placed in a 4 °C refrigerator in the dark for 24 h. The supernatant collected after extraction was the chlorophyll extract. Then, the chlorophyll concentrations were determined by detecting the absorbance at 665 nm and 649 nm using a UV2400 UV/VIS spectrophotometer (Soptpo, Shanghai, China) as previously described [55].

Measurements of relative ion leakage rate were obtained using a bench-top conductivity meter (DDS 307A, Jingke Industrial, Shanghai, China). The leaves were cut into pieces and then added with 10 mL distilled water. After sealing, the leaves were oscillated at 180 rpm for 12 h at room temperature, and the detected conductivity was the initial conductivity (S1). Then, it was placed in a water bath and reacted at 100 °C for 15 min, and cooled to room temperature. The conductivity at this time was the final conductivity (S2). The relative electrical conductivity (REC) was calculated as follows: REC(%) = S1/S2 × 100 [40]. Three biological replications were carried out.

### 4.9. Subcellular Localization

The full-length coding region of *ZmSAG39* (without the stop codon) was inserted into the pCAMBIA1302 vector controlled by the 35S promoter to construct the GFP containing *ZmSAG39* vector (35S:ZmSAG39-GFP) (Figure S4). The recombinant plasmid and control vector (35S:GFP) were transferred into separate tobacco mesophyll cells. After 48 h, the specimens were observed under a laser scanning confocal microscope [56].

### 4.10. Yeast Two-Hybrid (Y2H)

The yeast two-hybrid vectors pGADT7-YKT62 and pGBKT7-ZmSAG39 were constructed and transformed into pairs in yeast strain AH109 (Figures S5 and S6). The transformed yeast competent cells were grown on culture medium (SD/-Leu-Trp). After the single colony grew, single colonies were selected and cultured to OD600 = 0.8, bacteria solution was grown on culture medium (SD/-Trp/-Leu, SD/-Trp/-Leu/-Ade/-His, SD/-Trp/-Leu/-Ade/-His containing X-α-Gal) at 29 °C constant temperature for 5–7 days and the colonies were observed [57].

### 4.11. qRT-PCR

The Trizol method was used to extract RNA from maize leaves, and the first strand of cDNA was synthesized using M-MLV Reverse Transcriptase (Invitrogen, Shanghai, China) following the manufacturer’s protocol. qRT-PCR was performed using SYBR PreMix Ex Taq (Takara, Changchun, China). The PCR program was: 95 °C for 10 min, followed by 40 cycles of 95 °C for 10 s, 60 °C for 20 s, and 72 °C for 15 s, followed by melting curve analysis [40]. The 2^−ΔΔCT^ method was used for data analysis. The *ZmACTIN1* gene was used as an internal reference to calculate Ct. Three biological replicates were performed for each sample. The primers used in this study are listed in Table S2.

### 4.12. Statistical Analysis

All results in this study were performed with three replicates. The data are presented as mean ± SD (standard deviation) of three biological replicates. Student’s *t*-test was used to confirm the variability of results between treatments. *p* < 0.05 (*) and *p* < 0.01 (**). The figures were prepared with GraphPad Prism 8 (V8.4.3, GraphPad, Changchun, China).

## Data Availability

The data presented in this study are available on request from the corresponding author.

## Supplementary Materials

The following supporting information can be downloaded at: https://www.mdpi.com/article/10.3390/ijms232415984/s1.
