# Supplementary material for: A Novel Senescence-Specific Gene (ZmSAG39) Negatively Regulates Darkness and Drought Responses in Maize
Source: Int J Mol Sci. 2022 Dec 15;23(24):15984. doi: 10.3390/ijms232415984 (PMC9785699; doi:10.3390/ijms232415984)
Supplement: Supplementary file 1 [file ijms-23-15984-s001.zip › ijms-2041993-supplementary.pdf]

Supplementary materials

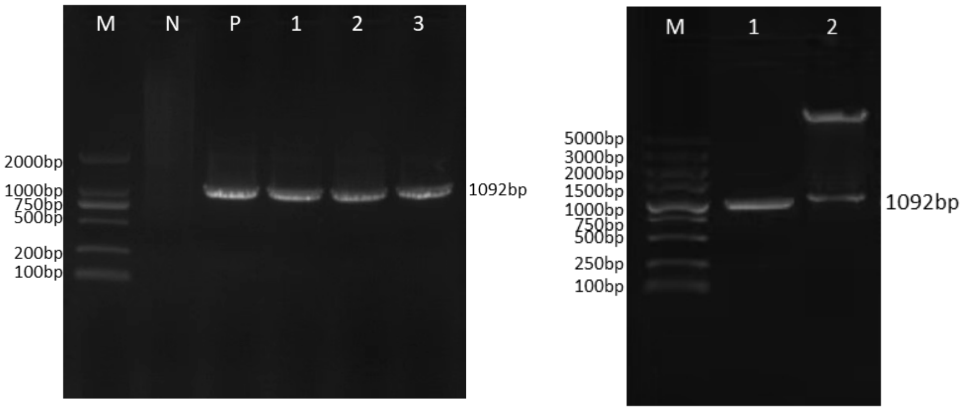

(**A**) (**B**)

Figure S1. Construction of the pCAMBIA3301-ZmSAG39 vector. (**A**) *ZmSAG39* gene test results. M: DNA Marker DL 2000; N: Blank control; P: Positive control; 1-3: PCR amplification results. (**B**) Double digestion verification results. M: DNA Marker DL 5000; 1: pCAMBIA3301-ZmSAG39 recombinant plasmid without restriction digestion; 2: pCAMBIA3301-ZmSAG39 recombinant plasmid with restriction digestion.

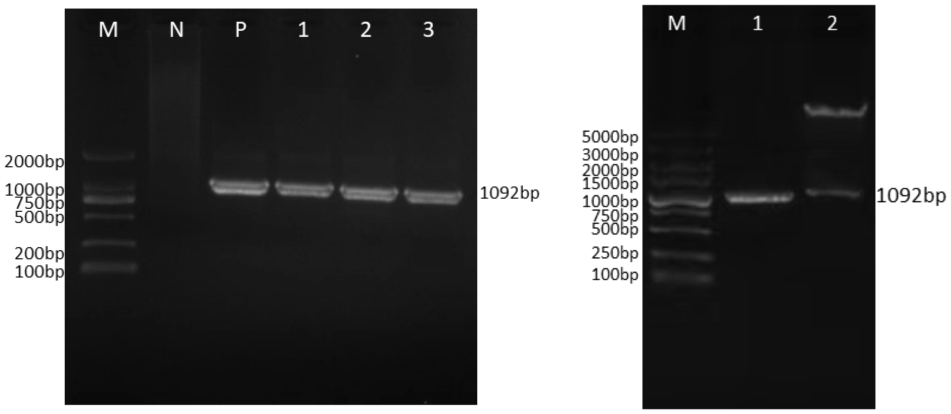

(**A**) (**B**)

Figure S2. Construction of the pCAMBIA1302-ZmSAG39 vector. (**A**) *ZmSAG39* gene test results. M: DNA Marker DL 2000; N: Negative control; P: Positive control; 1-3: PCR amplification results. (**B**) Double digestion verification results. M: DNA Marker DL 5000; 1: pCAMBIA1302-ZmSAG39 recombinant plasmid without restriction digestion; 2: pCAMBIA1302-ZmSAG39 recombinant plasmid with restriction digestion.

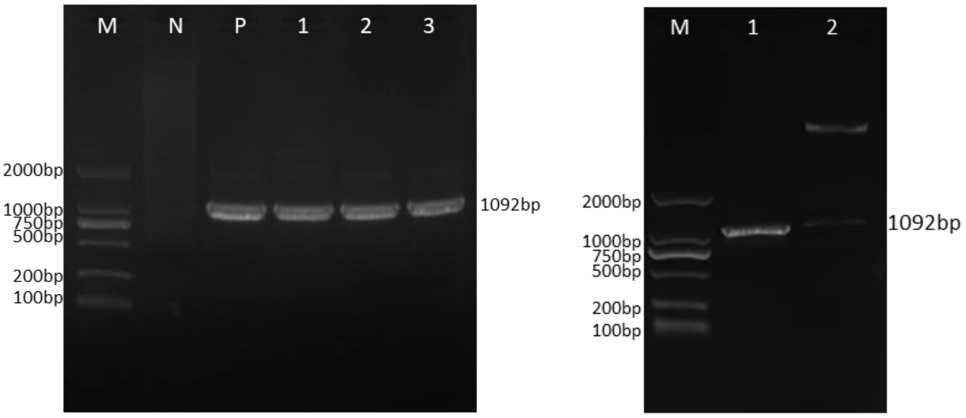

(**A**) (**B**)

Figure S3. Construction of the pGBKT7-ZmSAG39 vector. (**A**) *ZmSAG39* gene test results. M: DNA Marker DL 2000; N: Negative control; P: Positive control; 1-3: PCR amplification results. (**B**) Double

digestion verification results. M: DNA Marker DL 5000; 1: pGBKT7-ZmSAG39 recombinant plasmid without restriction digestion; 2: pGBKT7-ZmSAG39 recombinant plasmid with restriction digestion.

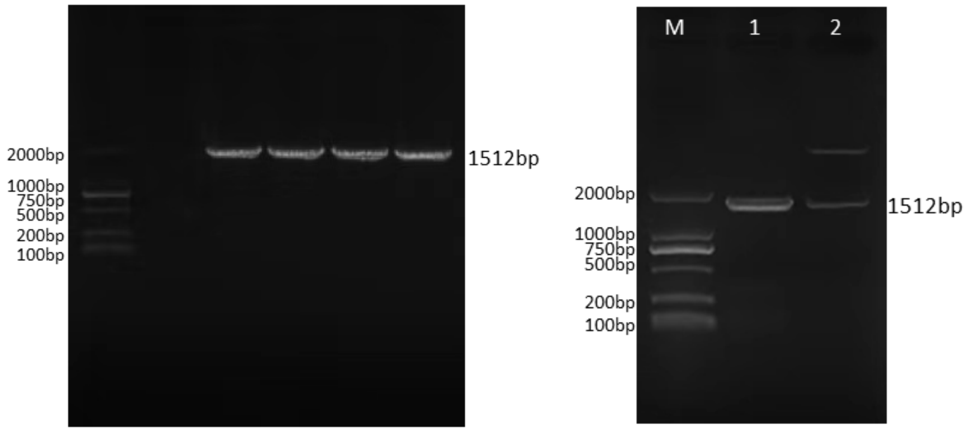

(**A**) (**B**)

Figure S4. Construction of the pGADT7-ZmYKT62 vector. (**A**) *ZmSAG39* gene test results. M: DNA Marker DL 2000; N: Negative control; P: Positive control; 1-3: PCR amplification results. (**B**) Double digestion verification results. M: DNA Marker DL 2000; 1: pGADT7-ZmYKT62 recombinant plasmid without restriction digestion; 2: pGADT7-ZmYKT62 recombinant plasmid with restriction digestion.

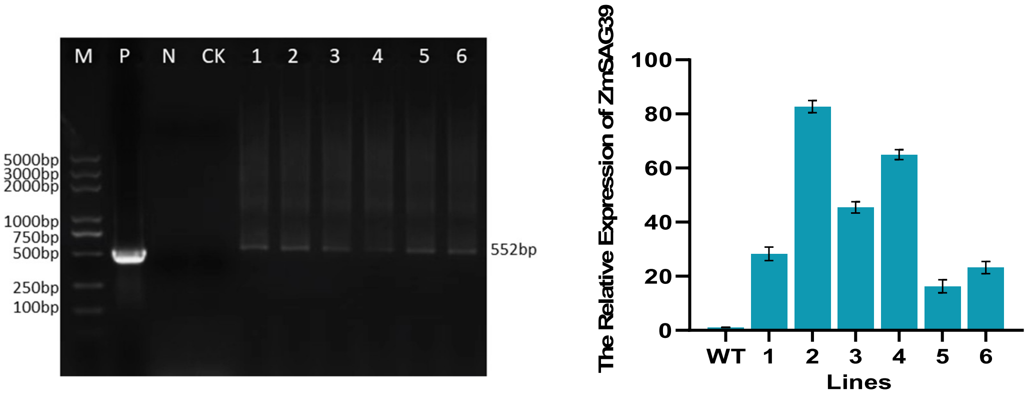

(**A**) (**B**)

Figure S5. Detection of *ZmSAG39* transgenic plants. (**A**)The overexpression of *ZmSAG39* gene in T2 transgenic maize was detected by RT –PCR. M: DNA Marker DL 5000; N: Negative control; P: Positive control; CK: Wild type plant; 1-6: PCR amplification results. (**B**) Expression analysis of ZmSAG39 in overexpression of ZmSAG39 lines. 1-6: Transgenic plant lines (OE3/OE7/OE8/OE11/0E15/0E21).

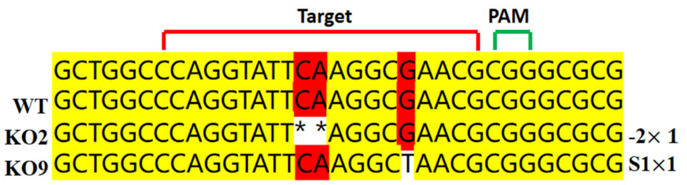

Figure S6. Sequences analysis revealed efficient targeted genome editing using the CRISPR-Cas9 system. The white letters in the sequence indicated mutations, where S indicated replacement and − indicated deletion. The letter on the right indicated the number of transgenic plants that were mutated in the same type of transgenic line.

Table S1. Primers for RT-PCR.

Primers Name	Primers Sequence(5'–3')
35S::ZmSAG39-F	acgacggccagtgccaagcttATGGGTCTCTGTCGAGTTCAATGTT

35S::ZmSAG39-R	acgggggactcttgaccatggGGAGCACCCGTGGATTCCTT
GFP-F	acgggggactcttgaccatggATGGGTCTCTGTCGAGTTCAATGTT
GFP-R	tctcctttactagtcagatctGGAGCACCCGTGGATTCCTT
BK-F	atggccatggaggccgaattcATGGGTCTCTGTCGAGTTCAATGTT
BK-R	ccgctgcaggtcgacggatccGGAGCACCCGTGGATTCCTT
AD-F	atggccatggaggccgaattcAACGCACGAAACCTCATT
AD-R	ccgctgcaggtcgacggatccACTGATAACAGAGTTCATTGCC
ZmSAG39-UP	GGCGGCTAGAGGAATGGTAC
ZmSAG39-LOW	ACTCTTTGCTGGTCAGGTCG

Table S2. Primers for qRT-PCR.

Primers Name	Primers Sequence(5'–3')
ZmMir3-F	GCAAGCTTCACGTTCTGCAA
ZmMir3-R	TGTATGTCCCGTTTCACGCA
ZmSee1-F	ACGCGCGAATTCCATTTCAG
ZmSee1-R	TCGAGCACGTAGAGGAGGAA
ZmCAO1-F	TCGAGGAGCAGTGGGTGATA
ZmCAO1-R	CATGGCAATGACTGGATGCG
ZmSAG39-F	ACATGAACCATGCAGTGACG
ZmSAG39-R	AGCTGCATGAAACCGTTCTC
ZmLTP3-F	ATGGCTGCTCCGAAGCTC
ZmLTP3-R	TGCAGTTAACGTTGGTGC
ZmSOS1-F	TGTTGCGTCACTTTGGGTAT
ZmSOS1-R	TCCTCGT-CATCCCTTAGTTC
ZmNYC1-F	ACGGGGGAGAGTACCAGTAC
ZmNYC1-R	ATTTGGCTGTCTCCACCTGG
ZmPAO-F	CCGTGGCAGAACTCTGTTCT
ZmPAO-R	GATGAGGCGAGGAAGGGATG
ZmActin1-F	ATGTTTCCTCCCATTGCCGAT
ZmActin1-R	CCAGTTTCGTCATACTCTCCCTTG
ZmRD20-F	CTCCTTTGCTGTCCATCCGT
ZmRD20-R	TGAGCTCAACTCGTCTTTTGTT
ZmSAG12-F	AGTTCGACGACAGCTTCTGG
ZmSAG12-R	GGAATTTGGAAGAGGCCGGA
